# Supplementary figures and images for: A comprehensive molecular characterization of the 8q22.2 region reveals the prognostic relevance of OSR2 mRNA in muscle invasive bladder cancer
Source: PLoS One. 2021 Mar 12;16(3):e0248342. doi: 10.1371/journal.pone.0248342 (PMC7954304; doi:10.1371/journal.pone.0248342)

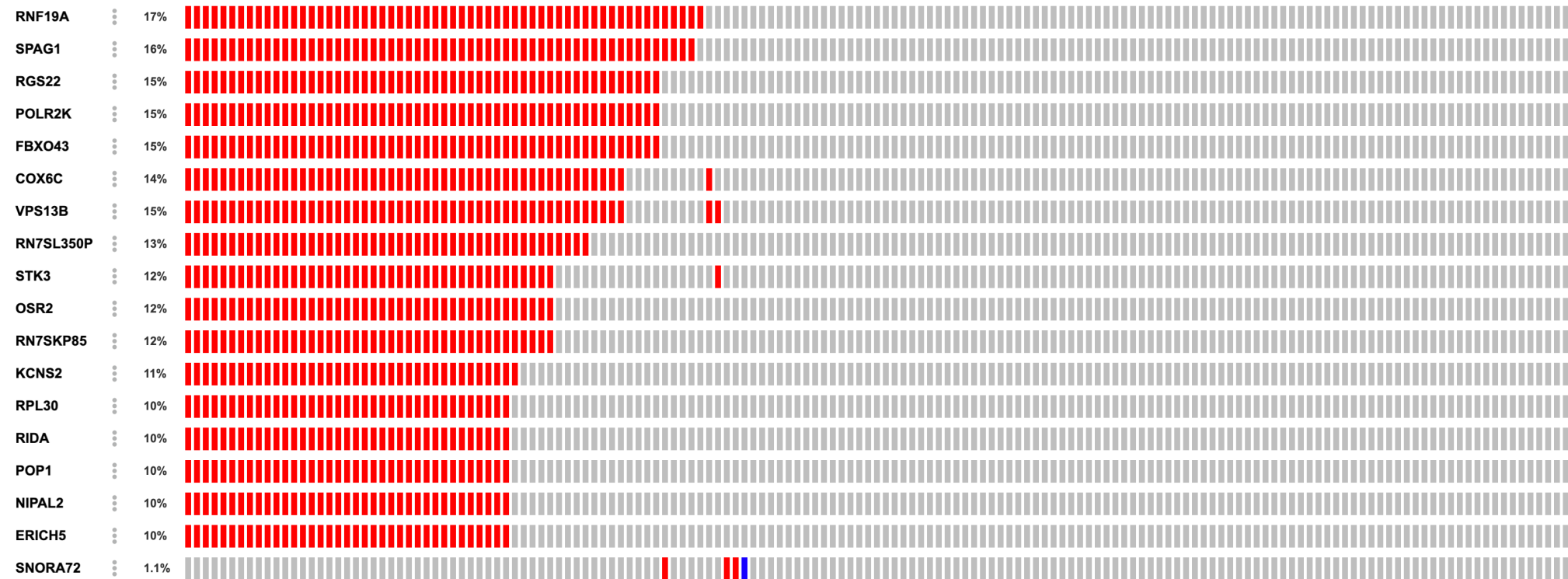

Supplement: S1 Fig — Red bars show copy number amplifications according to GISTIC2.0. Blue bars represent deep deletions. RN7SL350P, RN7SKP85 and SNORA72 were excluded from further analysis due to a lack of mRNA gene expression data. The graphic was created using cBioPortal’s oncoprint tool. (PDF) [file pone.0248342.s001.pdf]

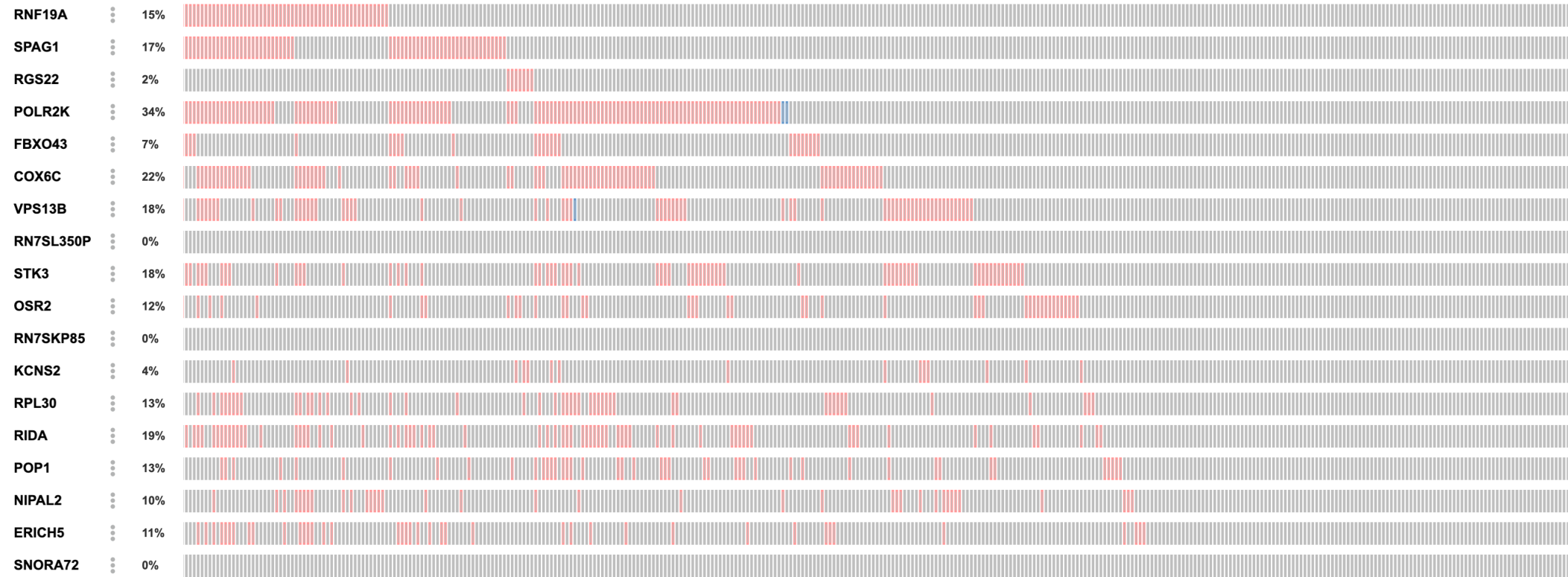

Supplement: S2 Fig — Red frames represent mRNA overexpression (z-score ≥2) and blue frames show low mRNA expression. RN7SL350P, RN7SKP85 and SNORA72 were excluded from further analysis due to a lack of mRNA gene expression data. The graphic was created using cBioPortal’s oncoprint tool. (PDF) [file pone.0248342.s002.pdf]

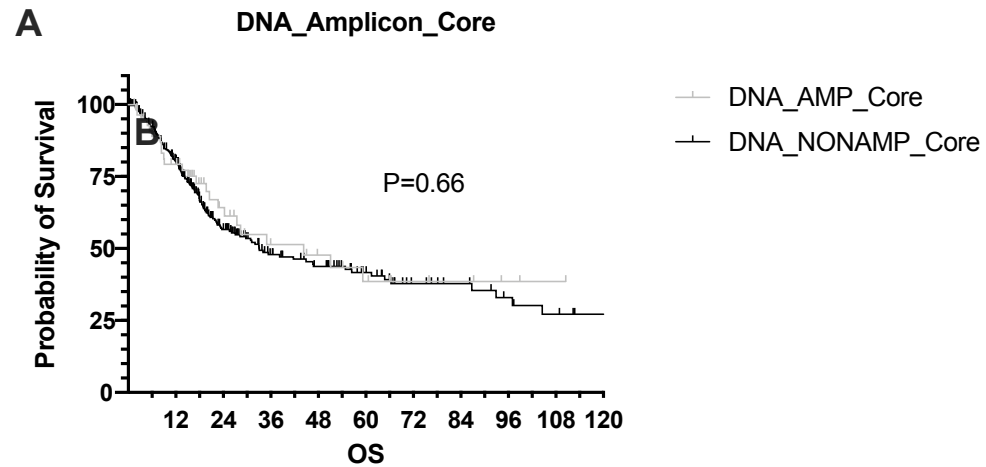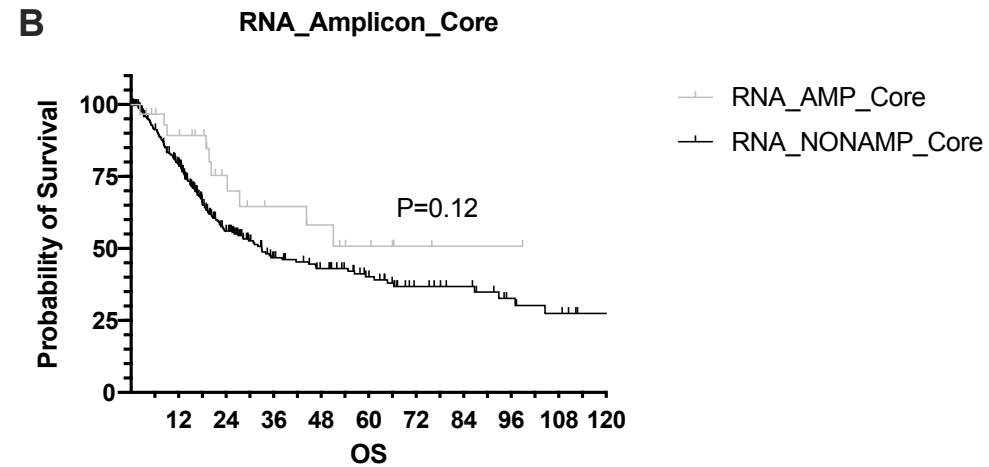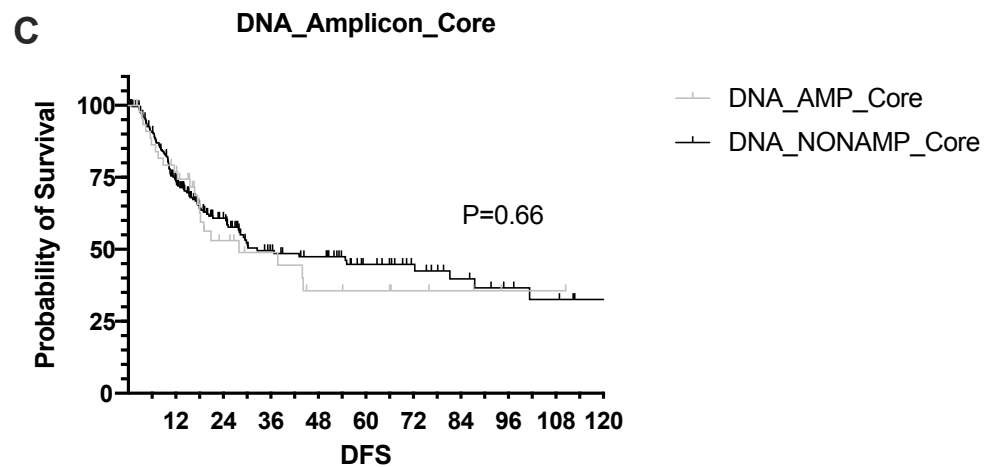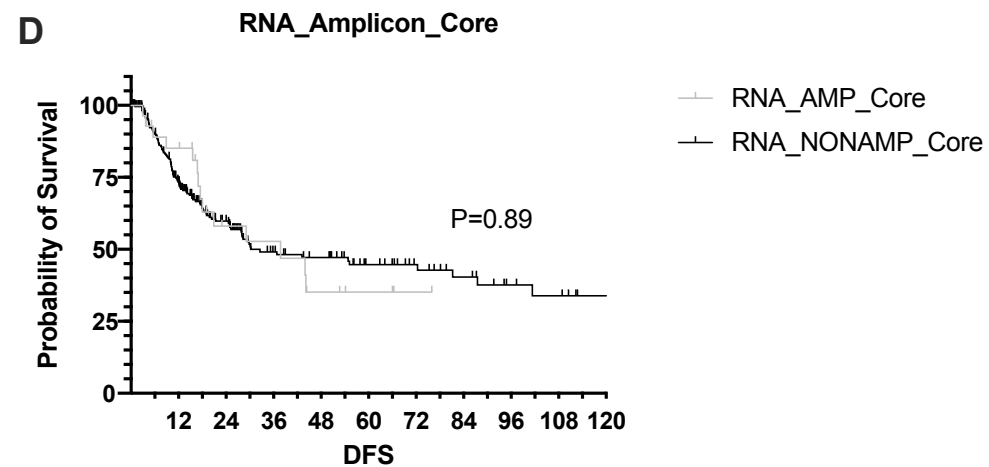

Supplement: S3 Fig — (A) Kaplan-Meier regression showing overall survival (OS) of patients with GISTIC2 core amplicons (AMP) vs. patients without GISTIC2 core amplicons (NONAMP). (B) Kaplan-Meier regression showing overall-survival (OS) of patients with mRNAz2 core amplicons (AMP) vs. patients without mRNAz2 core amplicons (NONAMP). (C) Kaplan-Meier regression showing disease-free survival (DFS) of patients with GISTIC2 core amplicons (AMP) vs. patients without GISTIC2 core amplicons (NONAMP). (D) Kaplan-Meier regression showing disease-free survival (DFS) of patients with mRNAz2 core amplicons (AMP) vs. patients without mRNAz2 core amplicons (NONAMP). AMP and NONAMP are shown in black and grey, respectively. No statistical differences between groups were observed. (5-year OS: AMP vs. NONAMP, p-value; DNA_Amplicon_Core: 39% vs. 42%; p = 0.66; RNA_Amplicon_Core: 51% vs. 40%; p = 0.12; DNA_Amplicon_Ext1: 46% vs 40%; p = 0.91; DNA_Amplicon_Ext2: 49% vs. 40%; p = 0.3)(5-Year DFS: AMP vs. NONAMP, p-value; DNA_Amplicon_Core: 36% vs. 45%; p = 0.66; RNA_Amplicon_Core: 35% vs. 45%; p = 0.89; DNA_Amplicon_Ext1: 41% vs 43%; p = 0.73; DNA_Amplicon_Ext2: 46% vs. 43%; p = 0.86). (PDF) [file pone.0248342.s003.pdf]

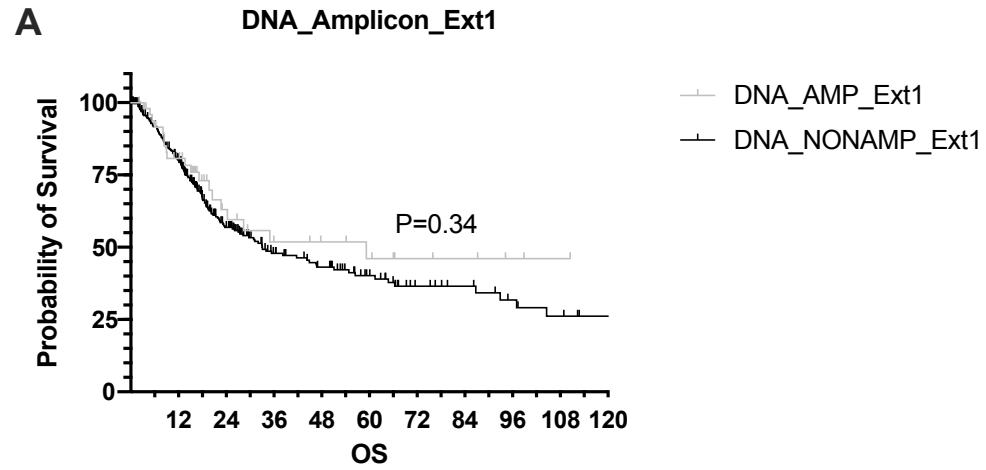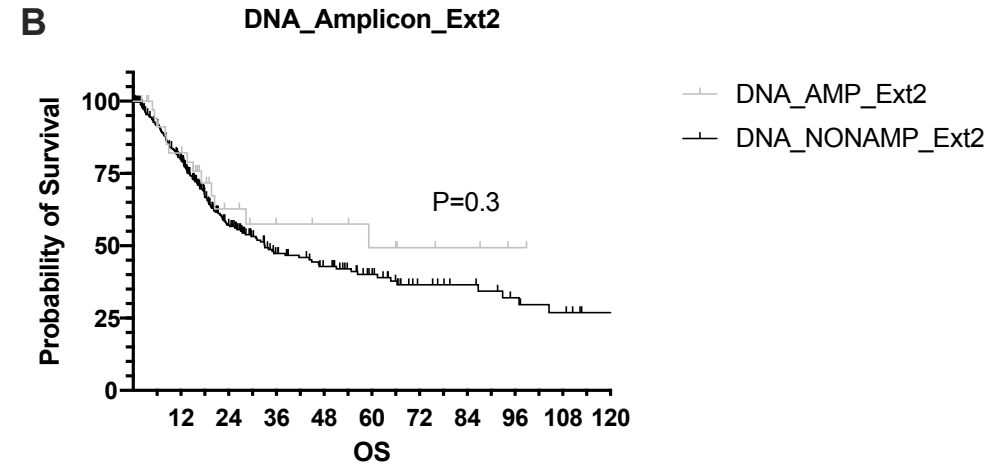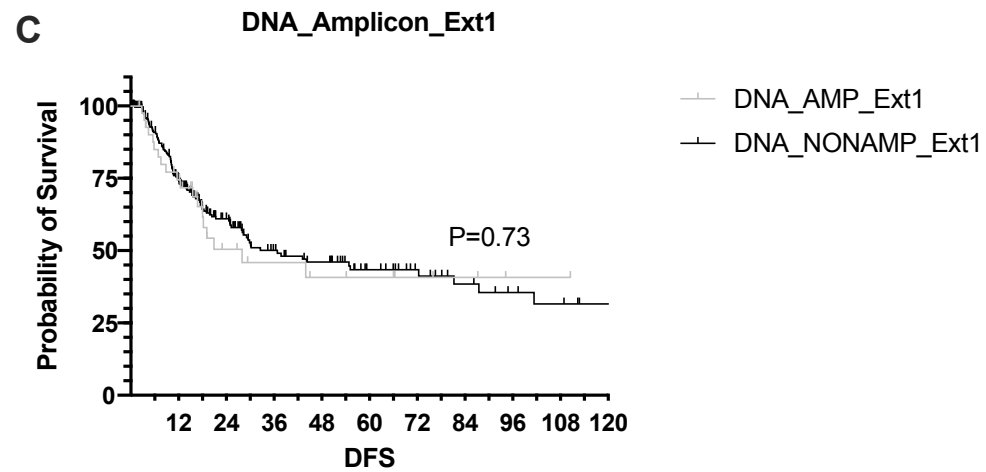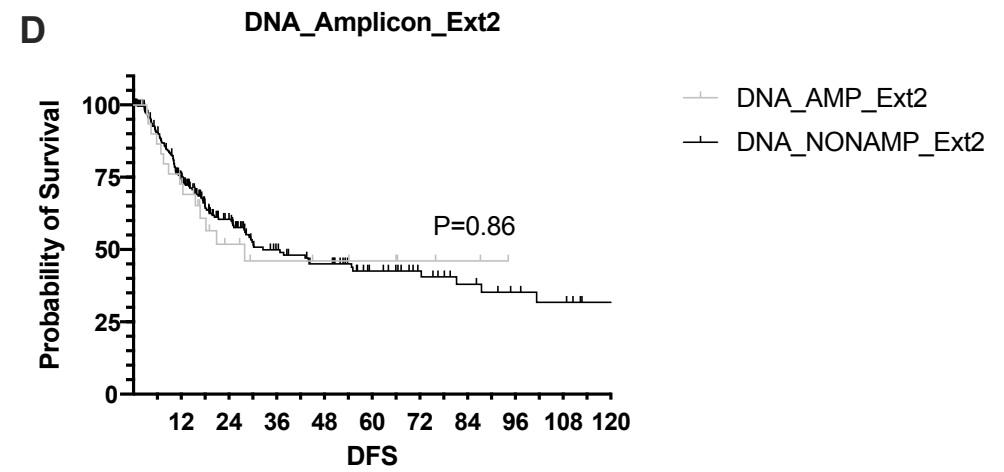

Supplement: S4 Fig — (A) Kaplan-Meier regression showing disease-free survival (DFS) of patients with GISTIC2 extended 1 amplicons (AMP) vs. patients without GISTIC2 extended 1 amplicons (NONAMP). (B) Kaplan-Meier regression showing disease-free survival (DFS) of patients with GISTIC2 extended 1 amplicons (AMP) vs. patients without GISTIC2 extended 1 amplicons (NONAMP). (C) Kaplan-Meier regression showing overall survival (OS) of patients with GISTIC2 extended 1 amplicons (AMP) vs. patients without GISTIC2 core amplicons (NONAMP). (D) Kaplan-Meier regression showing overall survival (OS) of patients with GISTIC2 extended 2 amplicons (AMP) vs. patients without GISTIC2 extended 2 amplicons (NONAMP). AMP and NONAMP are shown in black and grey, respectively. No statistical differences between groups were observed. (PDF) [file pone.0248342.s004.pdf]

Distribution of amplified genes according to amplicon definition

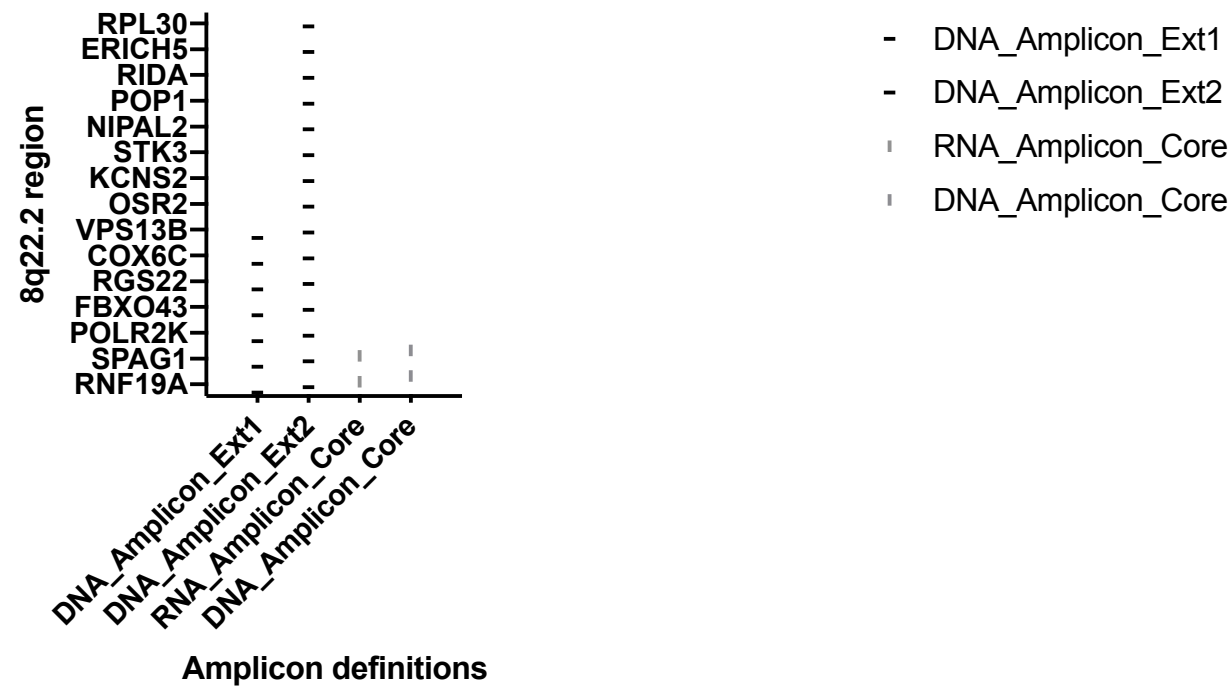

Supplement: S6 Fig — Genes of the 8q22.2 region are arranged according to their genomic location and distributed accross amplicon definitions based on the extent of the percentage of coamplification. DNA_Amplicon_Ext1 and DNA_Amplicon_Ext2 extend beyond the core region of RNF19A and SPAG1, to include seven genes from RNF19A to VPS13B (DNA_Amplicon_Ext1) and 15 genes from RNF19A to ERICH5 (DNA_Amplicon_Ext2). (PDF) [file pone.0248342.s006.pdf]

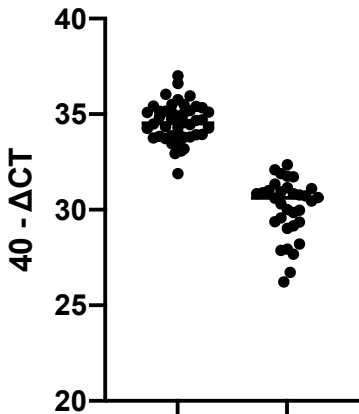

40-delta CT COX6C Mean

40-delta CT OSR2 Mean

normalized gene expression

Supplement: S7 Fig — Distribution of normalized 40-ΔCt values for COX6C and OSR2. Median gene expression was compared using the Mann-Whitney test and was significantly different between both genes (COX6C: 30.62; OSR2: 34,53; p<0.001). (PDF) [file pone.0248342.s007.pdf]
