# Supplementary material for: A comprehensive molecular characterization of the 8q22.2 region reveals the prognostic relevance of OSR2 mRNA in muscle invasive bladder cancer
Source: PLoS One. 2021 Mar 12;16(3):e0248342. doi: 10.1371/journal.pone.0248342 (PMC7954304; doi:10.1371/journal.pone.0248342)
Supplement: S5 Fig — Distribution of lymph node status (%) is stratified according to 4 combinations of copy number amplification or deletion and high (z-score ≥ 2) or low (z-score < 2) mRNA expression. Lymph node positive and negative stage are almost evenly distributed for the combination of amplification and overexpression, while all other combinations show a greater proportion of negative lymph node stage. (PDF) [file pone.0248342.s005.pdf]

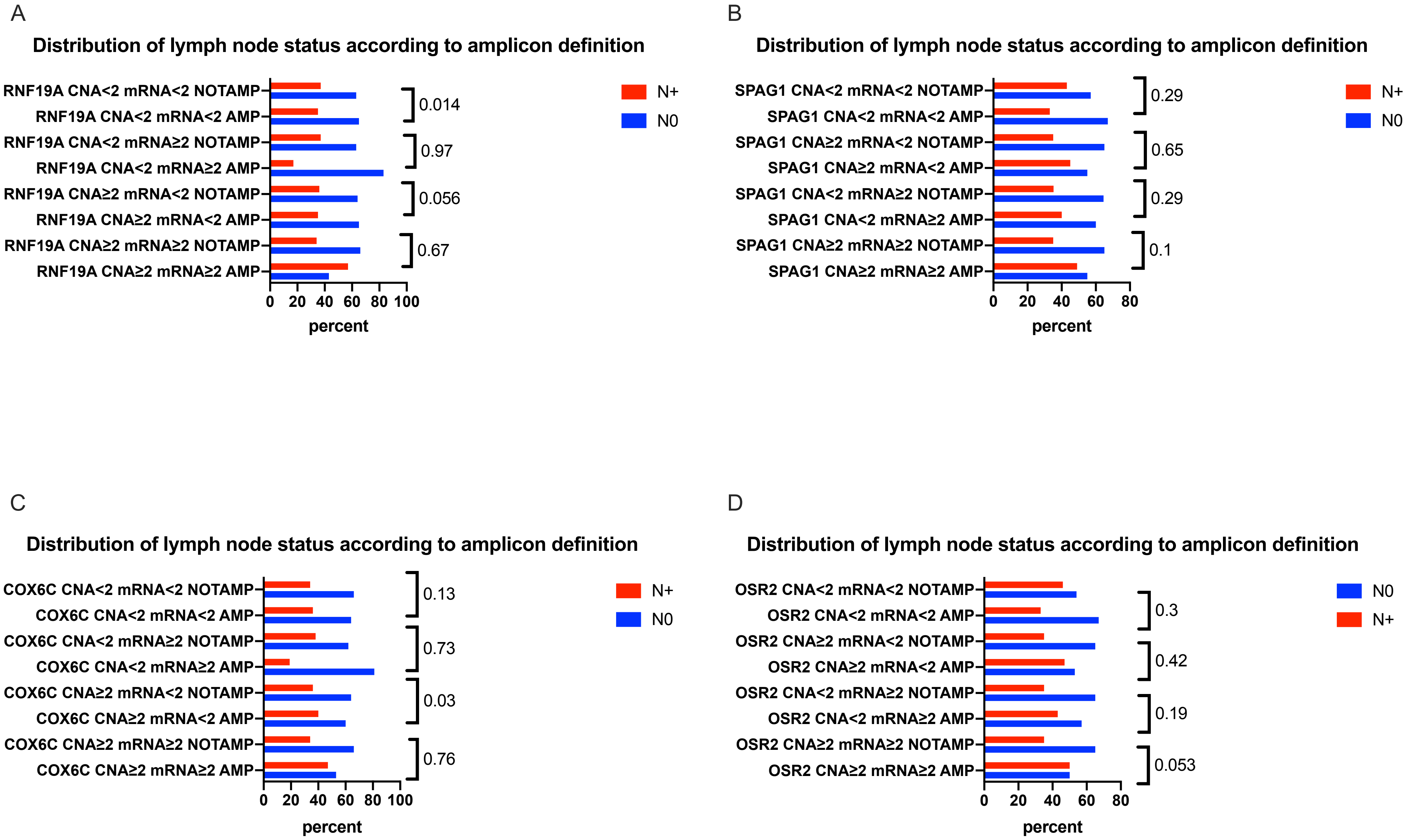

A
Distribution of lymph node status according to amplicon definition
RNF19A CNA<2 mRNA<2 NOTAMP
RNF19A CNA<2 mRNA<2 AMP
RNF19A CNA<2 mRNA≥2 NOTAMP
RNF19A CNA<2 mRNA≥2 AMP
RNF19A CNA≥2 mRNA<2 NOTAMP
RNF19A CNA≥2 mRNA<2 AMP
RNF19A CNA≥2 mRNA≥2 NOTAMP
RNF19A CNA≥2 mRNA≥2 AMP
0.014
0.97
0.056
0.67
0 20 40 60 80 100
percent
N+
N0
B
Distribution of lymph node status according to amplicon definition
SPAG1 CNA<2 mRNA<2 NOTAMP
SPAG1 CNA<2 mRNA<2 AMP
SPAG1 CNA≥2 mRNA<2 NOTAMP
SPAG1 CNA≥2 mRNA<2 AMP
SPAG1 CNA<2 mRNA≥2 NOTAMP
SPAG1 CNA<2 mRNA≥2 AMP
SPAG1 CNA≥2 mRNA≥2 NOTAMP
SPAG1 CNA≥2 mRNA≥2 AMP
0.29
0.65
0.29
0.1
0 20 40 60 80
percent
N+
N0
C
Distribution of lymph node status according to amplicon definition
COX6C CNA<2 mRNA<2 NOTAMP
COX6C CNA<2 mRNA<2 AMP
COX6C CNA<2 mRNA≥2 NOTAMP
COX6C CNA<2 mRNA≥2 AMP
COX6C CNA≥2 mRNA<2 NOTAMP
COX6C CNA≥2 mRNA<2 AMP
COX6C CNA≥2 mRNA≥2 NOTAMP
COX6C CNA≥2 mRNA≥2 AMP
0.13
0.73
0.03
0.76
0 20 40 60 80 100
percent
N+
N0
D
Distribution of lymph node status according to amplicon definition
OSR2 CNA<2 mRNA<2 NOTAMP
OSR2 CNA<2 mRNA<2 AMP
OSR2 CNA≥2 mRNA<2 NOTAMP
OSR2 CNA≥2 mRNA<2 AMP
OSR2 CNA<2 mRNA≥2 NOTAMP
OSR2 CNA<2 mRNA≥2 AMP
OSR2 CNA≥2 mRNA≥2 NOTAMP
OSR2 CNA≥2 mRNA≥2 AMP
0.3
0.42
0.19
0.053
0 20 40 60 80
percent
N0
N+
