# Supplementary material for: A comprehensive molecular characterization of the 8q22.2 region reveals the prognostic relevance of OSR2 mRNA in muscle invasive bladder cancer
Source: PLoS One. 2021 Mar 12;16(3):e0248342. doi: 10.1371/journal.pone.0248342 (PMC7954304; doi:10.1371/journal.pone.0248342)
Supplement: S1 Table — (DOCX) [file pone.0248342.s010.docx]

S1 Table. Univariable analysis of mRNA amplicon definitions with different z-score cut-offs.

| z-score | Overall survival | | Disease-free survival | |
| --- | --- | --- | --- | --- |
|  | HR | P-value | HR | P-value |
| **1** | **0.66 [0.42; 1.01]** | **0.046** | 0.93 [0.6; 1.43] | 0.73 |
| 2 | 0.6 [0.32; 1.14] | 0.12 | 1.04 [0.59; 1.82] | 0.88 |
| 3 | 0.72 [0.35; 1.46] | 0.36 | 1.1 [0.58; 2.1] | 0.77 |
