# Supplementary material for: A comprehensive molecular characterization of the 8q22.2 region reveals the prognostic relevance of OSR2 mRNA in muscle invasive bladder cancer
Source: PLoS One. 2021 Mar 12;16(3):e0248342. doi: 10.1371/journal.pone.0248342 (PMC7954304; doi:10.1371/journal.pone.0248342)
Supplement: S2 Table — (DOCX) [file pone.0248342.s011.docx]

S2 Table. Multivariable analysis of Overall survival of mRNA amplicon with z-score cut-off z=1.

| **Clinicopathologic variables** |  | Overall survival | |
| --- | --- | --- | --- |
|  |  | HR | P-value |
|  |  |  |  |
| Age | ≥70 vs <70 | 1.76 [1.24; 2.49] | 0.0015 |
| T stage | T3/4 vs T≤2 | 1.5 [0.98; 2.32] | 0.065 |
| Lymph node status | N+ vs N0 | 2.05 [1.44; 2.91] | <0.001 |
| Molecular subtype | (basal vs. luminal) | 1.2 [0.84; 1.7] | 0.32 |
| RNA_Amplicon_Core_z2 | AMP vs NONAMP | 0.65 [0.28; 1.52] | 0.32 |
| RNA_Amplicon_Core_z1 | AMP vs NONAMP | 0.98 [0.54; 1.77] | 0.94 |
| RNA_AMP_COX6C | HIGH vs LOW | 1.31 [0.8; 2.14] | 0.29 |
| RNA_AMP_OSR2 | HIGH vs LOW | 0.71 [0.43; 1.16] | 0.17 |
