# Supplementary material for: A comprehensive molecular characterization of the 8q22.2 region reveals the prognostic relevance of OSR2 mRNA in muscle invasive bladder cancer
Source: PLoS One. 2021 Mar 12;16(3):e0248342. doi: 10.1371/journal.pone.0248342 (PMC7954304; doi:10.1371/journal.pone.0248342)
Supplement: S3 Table — (DOCX) [file pone.0248342.s012.docx]

S3 Table. Median gene expression of the core CNA amplicon in AMP and NAMP

| **Genes** | **Median AMP (CNA)** | **Median NONAMP (CNA)** | **P-value** | **P-value (Bonferroni adjusted)** |
| --- | --- | --- | --- | --- |
| RNF19A | 1.91 | 0.028 | 5e-10 | 7.5e-9 |
| SPAG1 | 1.94 | 0.11 | 6e-12 | 9e-11 |
| RGS22 | -0.28 | -0.26 | 0.89 | 1. |
| POLR2K | 5.84 | 0.78 | 2e-22 | <0.001 |
| FBXO43 | 0.3 | -0.17 | 0.0002 | 0.003 |
| COX6C | 2.9 | 0.09 | 4e-16 | 6e-15 |
| VPS13B | 1.13 | 0.13 | 0.0003 | 0.0045 |
| RN7SL350P |  |  |  |  |
| STK3 | 1.59 | 0.41 | 7.1e-6 | 0.0001 |
| OSR2 | 0.22 | -0.18 | 0.018 | 0.27 |
| RN7SKP85 |  |  |  |  |
| KCNS2 | -0.22 | -0.31 | 0.11 | 1. |
| RPL30 | 1.35 | -0.002 | 2.2e-8 | 3.3e-7 |
| RIDA | 2.25 | 0.2 | 5e-14 | 7.5e-13 |
| POP1 | 0.36 | -0.1 | 0.0003 | 0.0045 |
| NIPAL2 | -0.22 | -0.08 | 0.77 | 1. |
| ERICH5 | 0.75 | -0.4 | 2.2e-6 | <0.001 |
| SNORA72 |  |  |  |  |
