# Supplementary material for: A comprehensive molecular characterization of the 8q22.2 region reveals the prognostic relevance of OSR2 mRNA in muscle invasive bladder cancer
Source: PLoS One. 2021 Mar 12;16(3):e0248342. doi: 10.1371/journal.pone.0248342 (PMC7954304; doi:10.1371/journal.pone.0248342)
Supplement: S4 Table — (DOCX) [file pone.0248342.s013.docx]

S4 Table. Subgroup analysis of molecular subtypes in the TCGA cohort.

| molecular subtype | **DNA_Amplicon_Core**  **(% of Amplified and Nonamplified patients with respective subtype)** | | P-value | **RNA_Amplicon_ Core**  **(% of Amplified and Nonamplified patients with respective subtype)** | | P-value |
| --- | --- | --- | --- | --- | --- | --- |
|  | AMP | NONAMP |  | AMP | NONAMP |  |
| luminal | 71 | 57 | **0.04** | 83 | 57 | **0.004** |
| basal squamous | 24 | 39 | **0.03** | 17 | 39 | **0.02** |
| Luminal papillary | 26 | 34 | 0.24 | 50 | 31 | **0.03** |
| Luminal infiltrated | 21 | 19 | 0.77 | 10 | 20 | 0.18 |
| Neuronal | 5 | 4 | 0.69 | 0 | 5 | 0.23 |
