# Supplementary material for: A comprehensive molecular characterization of the 8q22.2 region reveals the prognostic relevance of OSR2 mRNA in muscle invasive bladder cancer
Source: PLoS One. 2021 Mar 12;16(3):e0248342. doi: 10.1371/journal.pone.0248342 (PMC7954304; doi:10.1371/journal.pone.0248342)
Supplement: S5 Table — (DOCX) [file pone.0248342.s014.docx]

S5 Table. Functions of genes in the 8q22.2 region.

| **Gene** | **Description** | **Function** |
| --- | --- | --- |
| **RNF19A** | E3 ubiquitin-protein ligase | ubiquitination |
| **SPAG1** | Sperm-associated antigen 1 | Axonemal dynein complex assembly |
| **RGS22** | Regulator of G-protein signaling 22 | Negative regulation of signal transduction |
| **POLR2K** | DNA-directed RNA. polymerases I, II, and III subunit RPABC4 | Transcription of DNA into RNA |
| **FBXO43** | F-box only protein 43 | Protein ubiquitination |
| **COX6C** | Cytochrome c oxidase subunit 6C | Mitochondrial electron transport |
| **VPS13B** | Vacuolar protein sorting-associated protein 13B | Protein transport and sorting |
| **RN7SL350P** | Pseudogene |  |
| **STK3** | Serine/threonine-protein kinase 3 | Pro-apoptotic kinase; Hippo signaling pathway |
| **OSR2** | Protein odd-skipped-related 2 | Embryonic morphogenesis |
| **RN7SKP85** | Pseudogene |  |
| **KCNS2** | Potassium voltage-gated channel subfamily S member 2 | Modulates channel activity of KCNB1 and KCNB2 |
| **RPL30** | 60S ribosomal protein L30 | translation |
| **RIDA** | 2-iminobutanoate/2-iminopropanoate deaminase |  |
| **POP1** | Ribonucleases P/MRP protein subunit POP1 | tRNA processing |
| **NIPAL2** | NIPA-like protein 2 | Mg^2+^ ion transport |
| **ERICH5** | Glutamate-rich protein 5 |  |
| **SNORA72** | Small Nucleolar RNA, H/ACA Box 72 |  |

Full names and functions of individual genes of the 8q22.2 region. No definitive function could be found for the two pseudogenes RN7SL350P and RN7SKP85, as well as RIDA, ERICH5 and SNORA72.
