# Supplementary material for: A comprehensive molecular characterization of the 8q22.2 region reveals the prognostic relevance of OSR2 mRNA in muscle invasive bladder cancer
Source: PLoS One. 2021 Mar 12;16(3):e0248342. doi: 10.1371/journal.pone.0248342 (PMC7954304; doi:10.1371/journal.pone.0248342)
Supplement: S6 Table — (DOCX) [file pone.0248342.s015.docx]

S6 Table. Rate of mutations in AMP and NONAMP patients for DNA_Amplicon_Core.

| **Mutation** | **DNA_AMP_Core** | **DNA_NONAMP_Core** | **p value** | **p value (Bonferroni adjusted)** |
| --- | --- | --- | --- | --- |
| **TP53** | **65** | **46** | **0.008** | 0.144 |
| KMT2D | 30 | 27 | 0.62 | 1. |
| KDM5A | 4 | 5 | 0.65 | 1. |
| ARID1A | 30 | 23 | 0.34 | 1. |
| PIK3CA | 26 | 21 | 0.38 | 1. |
| KMT2C | 12 | 18 | 0.28 | 1. |
| RB1 | 11 | 19 | 0.11 | 1. |
| EP300 | 18 | 16 | 0.85 | 1. |
| **FGFR3** | **4** | **15** | **0.02** | **0.36** |
| STAG2 | 9 | 16 | 0.17 | 1. |
| ATM | 21 | 12 | 0.08 | 1. |
| FAT1 | 9 | 13 | 0.41 | 1. |
| ELF3 | 12 | 12 | 0.88 | 1. |
| **CREBBP** | **4** | **13** | **0.04** | 0.72 |
| **ERBB2** | **4** | **13** | **0.04** | 0.72 |
| SPTAN1 | 12 | 11 | 0.7 | 1. |
| **KMT2A** | **19** | **10** | **0.04** | **0.72** |
| ERBB3 | 7 | 9 | 0.61 | 1. |
