# Supplementary material for: A comprehensive molecular characterization of the 8q22.2 region reveals the prognostic relevance of OSR2 mRNA in muscle invasive bladder cancer
Source: PLoS One. 2021 Mar 12;16(3):e0248342. doi: 10.1371/journal.pone.0248342 (PMC7954304; doi:10.1371/journal.pone.0248342)
Supplement: S7 Table — (DOCX) [file pone.0248342.s016.docx]

S7 Table. Rate of mutations in AMP and NONAMP patients for RNA_Amplicon_Core.

| **Mutation** | **RNA_AMP_Core** | **RNA_NONAMP_Core** | **p value** | **p value (Bonferroni adjusted)** |
| --- | --- | --- | --- | --- |
| TP53 | 53 | 48 | 0.61 | 1. |
| KMT2D | 27 | 27 | 0.95 | 1. |
| KDM5A | 3 | 5 | 0.71 | 1. |
| ARID1A | 17 | 26 | 0.28 | 1. |
| PIK3CA | 23 | 22 | 0.85 | 1. |
| KMT2C | 17 | 17 | 0.93 | 1. |
| RB1 | 10 | 19 | 0.24 | 1. |
| EP300 | 13 | 17 | 0.61 | 1. |
| FGFR3 | 7 | 14 | 0.27 | 1. |
| STAG2 | 10 | 15 | 0.45 | 1. |
| ATM | 17 | 13 | 0.63 | 1. |
| FAT1 | 7 | 13 | 0.35 | 1. |
| ELF3 | 10 | 12 | 0.76 | 1. |
| CREBBP | 3 | 12 | 0.15 | 1. |
| **ERBB2** | **0** | **13** | **0.04** | **0.72** |
| SPTAN1 | 10 | 11 | 0.88 | 1. |
| KMT2A | 20 | 11 | 0.12 | 1. |
| ERBB3 | 7 | 9 | 0.67 | 1. |
