# Supplementary material for: A comprehensive molecular characterization of the 8q22.2 region reveals the prognostic relevance of OSR2 mRNA in muscle invasive bladder cancer
Source: PLoS One. 2021 Mar 12;16(3):e0248342. doi: 10.1371/journal.pone.0248342 (PMC7954304; doi:10.1371/journal.pone.0248342)
Supplement: S8 Table — (DOCX) [file pone.0248342.s017.docx]

S8 Table. Association of gains at 8q22.2 with other genomic events.

|  | Amplification | No amplification | P-value | Bonferroni adjusted |
| --- | --- | --- | --- | --- |
| **focal amplification** |  |  |  |  |
| **6p22.3** | **29** | **13** | **0.002** | **0.066** |
| **1q23.3** | **34** | **15** | **0.0004** | **0.0132** |
| **8q22.3** | **84** | **10** | **1.78e-36** | **<0.001** |
| 11q13.3 | 12 | 10 | 0.7 | 1. |
| **3p25.2** | **33** | **9** | **1.419e-6** | **<0.001** |
| 12q15 | 12 | 7 | 0.23 | 1. |
| 10p14 | 16 | 9 | 0.13 | 1. |
| 8p11.23 | 10 | 10 | 0.94 | 1. |
| **19q12** | **21** | **4** | **3.459e-6** | **0.0001** |
| 1p34.2 | 10 | 5 | 0.11 | 1. |
| 20q11.21 | 12 | 9 | 0.42 | 1. |
| 5p15.33 | 5 | 14 | 0.055 | 1. |
| 17q12 | 10 | 4 | 0.06 | 1. |
| **1q21.2** | **19** | **9** | **0.03** | **0.99** |
| 7p11.2 | 3 | 7 | 0.28 | 1. |
| **7p21.1** | **14** | **6** | **0.0499** | 1. |
| 9p24.1 | 3 | 2 | 0.62 | 1. |
| 4p16.3 | 2 | 3 | 0.67 | 1. |
| 12p12.1 | 2 | 4 | 0.35 | 1. |
| **focal deletions** |  |  |  |  |
| 9p21.3 | 34 | 44 | 0.16 | 1. |
| 13q14.2 | 17 | 15 | 0.72 | 1. |
| 5q12.1 | 26 | 22 | 0.49 | 1. |
| 4q22.1 | 19 | 15 | 0.496 | 1. |
| 16p13.3 | 21 | 12 | 0.09 | 1. |
| 16p23.1 | 16 | 13 | 0.61 | 1. |
| 2q22.2 | 14 | 19 | 0.37 | 1. |
| 10q23.31 | 9 | 14 | 0.29 | 1. |
| **1p36.11** | **10** | **3** | **0.01** | **0.33** |
| 17p11.2 | 28 | 20 | 0.22 | 1. |
| 14q24.1 | 16 | 10 | 0.22 | 1. |
| **3p14.2** | **22** | **9** | **0.004** | **0.132** |
| 9p23 | 22 | 27 | 0.49 | 1. |
| Xp11.3 | 2 | 6 | 0.18 | 1. |
