# Supplementary material for: A comprehensive molecular characterization of the 8q22.2 region reveals the prognostic relevance of OSR2 mRNA in muscle invasive bladder cancer
Source: PLoS One. 2021 Mar 12;16(3):e0248342. doi: 10.1371/journal.pone.0248342 (PMC7954304; doi:10.1371/journal.pone.0248342)
Supplement: S9 Table — (DOCX) [file pone.0248342.s018.docx]

S9 Table. Sequence of primers and probes used in the RT-qPCR

| Primer/Probe | Sequence | Annealing  temperature |
| --- | --- | --- |
| CALM2 Forward | GAGCGAGCTGAGTGGTTGTG | 61.4˚C |
| CALM2 Reverse | AGTCAGTTGGTCAGCCATGCT | 59.8˚C |
| CALM2 Probe | TCGCGTCTCGGAAACCGGTAGC | 65.8˚C |
| COX6C Forward | GACAGTAACTACCATGGCTCCCGA | 64.4°C |
| COX6C Reverse | GCCACACGAAACTTATACAAAGCTG | 61.3°C |
| COX6C Probe | TGCCAAAACCTCGGATGCGTGGCCTTCTGGC | 73.5°C |
| OSR2 Forward | ACACATGCAGGAATCTCCACAC | 60.3°C |
| OSR2 Reverse | TTTCGCCTGAACACTTTGCCGC | 62.1°C |
| OSR2 Probe | ACTGCTCGCAGCTGTAGGGCTTGATGTCTGTA | 70.8°C |
