# Supplementary material for: A comprehensive molecular characterization of the 8q22.2 region reveals the prognostic relevance of OSR2 mRNA in muscle invasive bladder cancer
Source: PLoS One. 2021 Mar 12;16(3):e0248342. doi: 10.1371/journal.pone.0248342 (PMC7954304; doi:10.1371/journal.pone.0248342)
Supplement: S10 Table — (DOCX) [file pone.0248342.s019.docx]

S10 Table. Spearman coefficient analysis of genes in the 8q22.2 region.

| **Gene 1** | **Gene 2** | **Spearman’s Rho** | **p value** |
| --- | --- | --- | --- |
| NIPAL2 | VPS13B | 0.69 | <0.001 |
| RPL30 | COX6C | 0.68 | <0.001 |
| SPAG1 | RNF19A | 0.64 | <0.001 |
| RIDA | POLR2K | 0.64 | <0.001 |
| COX6C | POLR2K | 0.63 | <0.001 |
| RIDA | COX6C | 0.54 | <0.001 |
| KCNS2 | RGS22 | 0.52 | <0.001 |
| POLR2K | SPAG1 | 0.5 | <0.001 |
| VPS13B | RNF19A | 0.5 | <0.001 |
| ERICH5 | POP1 | -0.19 | <0.001 |
| POP1 | RGS22 | -0.19 | <0.001 |
| ERICH5 | FBXO43 | -0.18 | <0.001 |
| ERICH5 | OSR2 | -0.15 | 0.004 |
| COX6C | RGS22 | -0.14 | 0.006 |
| ERICH5 | STK3 | -0.14 | 0.007 |
| RPL30 | RGS22 | -0.14 | 0.008 |
| KCNS2 | COX6C | -0.14 | 0.01 |
| NIPAL2 | COX6C | -0.14 | 0.011 |
| RPL30 | KCNS2 | -0.12 | 0.02 |

The core region (RNF19A and SPAG1) as well as *POLR2K*, *COX6C* and *RIDA2* are highly correlated, while multiple negative correlations around *COX6C* and *ERICH5* can be seen. All shown correlations are statistically significant.
