# Supplementary material for: A comprehensive molecular characterization of the 8q22.2 region reveals the prognostic relevance of OSR2 mRNA in muscle invasive bladder cancer
Source: PLoS One. 2021 Mar 12;16(3):e0248342. doi: 10.1371/journal.pone.0248342 (PMC7954304; doi:10.1371/journal.pone.0248342)
Supplement: S11 Table — (DOCX) [file pone.0248342.s020.docx]

S11 Table. Univariable OS and DFS analysis of COX6C and OSR2 in vitro in a cohort from the university hospital Mannheim (n=46).

|  |  | Univariable analysis | | | |
| --- | --- | --- | --- | --- | --- |
|  |  | Overall survival* | | Disease-free survival† | |
| COX6C | median | 2.6 [0.92; 7.29] | 0.07 | 3.05 [0.81; 11.52] | 0.099 |
|  | 3rd quartile | 4.49 [1.69; 11.91] | 0.0026 | 4.29 [1.28; 14.42] | 0.018 |
|  | 1st quartile | NA | NA | NA | NA |
| OSR2* | median | 6.25 [1.37; 28.38] | 0.0177 | 4.54 [0.93; 22.16] | 0.06 |
|  | 3rd quartile | 2.06 [0.69; 6.18] | 0.2 | 1.84 [0.49; 6.91] | 0.37 |
|  | 1st quartile | 3.31 [0.43; 25.5] | 0.25 | 2.35 [0.29; 18.87] | 0.42 |

*34 patients for OSR2 due to missing gene expression data for 12 patients

†33 patients for OSR2 due to missing gene expression data for 12 patients and missing disease-free survival data for one patient
