# Supplementary material for: A comprehensive molecular characterization of the 8q22.2 region reveals the prognostic relevance of OSR2 mRNA in muscle invasive bladder cancer
Source: PLoS One. 2021 Mar 12;16(3):e0248342. doi: 10.1371/journal.pone.0248342 (PMC7954304; doi:10.1371/journal.pone.0248342)
Supplement: S12 Table — (DOCX) [file pone.0248342.s021.docx]

S12 Table. Univariable analysis of clinicopathologic features of the cohort from the university hospital Mannheim (n=46 patients).

| Univariable analysis |  | OS (n=46) | | DFS (n=45) | |
| --- | --- | --- | --- | --- | --- |
| Age | ≥ 70 vs. < 70 | 1.15 [0.43; 3.07] | 0.78 | 1.03 [0.3; 3.52] | 0.97 |
| Gender | male vs. female | 0.51 [0.19; 1.37] | 0.18 | 0.46 [0.14; 1.58] | 0.22 |
| T stage | T3/4 vs. T2 | 8.14 [1.08; 61.67] | 0.04 | 2.3997e+9 | 0.9992 |
| N stage | N+ vs. N0 | 3.12 [1.07; 9.1] | 0.037 | 2.9 [0.81; 10.43] | 0.1 |
