# Supplementary material for: A comprehensive molecular characterization of the 8q22.2 region reveals the prognostic relevance of OSR2 mRNA in muscle invasive bladder cancer
Source: PLoS One. 2021 Mar 12;16(3):e0248342. doi: 10.1371/journal.pone.0248342 (PMC7954304; doi:10.1371/journal.pone.0248342)
Supplement: S13 Table — (DOCX) [file pone.0248342.s022.docx]

S13 Table. Multivariable analysis of OS for COX6C in the university hospital Mannheim cohort (n=46)

| COX6C multivariable | | OS | |
| --- | --- | --- | --- |
| T stage | T3/4 vs. T2 | 2.9228e+9 | 0.9991 |
| N stage | N+ vs. N0 | 2.94 [0.97; 8.91] | 0.056 |
| COX6C | median | 3.41 [1.05; 11.1] | 0.04 |
