# Supplementary material for: A comprehensive molecular characterization of the 8q22.2 region reveals the prognostic relevance of OSR2 mRNA in muscle invasive bladder cancer
Source: PLoS One. 2021 Mar 12;16(3):e0248342. doi: 10.1371/journal.pone.0248342 (PMC7954304; doi:10.1371/journal.pone.0248342)
Supplement: S14 Table — (DOCX) [file pone.0248342.s023.docx]

S14 Table. Univariable analysis of DFS for OSR2 in the university hospital Mannheim cohort (n=33)

| Univariable analysis for 34 patients |  | DFS (n=33) | |
| --- | --- | --- | --- |
| Age | ≥ 70 vs. < 70 | 1.11 [0.28; 4.49] | 0.88 |
| Gender | male vs. female | 0.73 [0.15; 3.53] | 0.7 |
| T stage | T3/4 vs. T2 | 879638035 |  |
| N stage | N+ vs. N0 | 3.2 [0.85; 12.12] | 0.09 |
