# Supplementary material for: A comprehensive molecular characterization of the 8q22.2 region reveals the prognostic relevance of OSR2 mRNA in muscle invasive bladder cancer
Source: PLoS One. 2021 Mar 12;16(3):e0248342. doi: 10.1371/journal.pone.0248342 (PMC7954304; doi:10.1371/journal.pone.0248342)
Supplement: S15 Table — (DOCX) [file pone.0248342.s024.docx]

S15 Table. Multivariable analysis of OS and DFS for OSR2 in the university hospital Mannheim cohort

| OSR2 multivariable (n=32) | | OS (n=32) | | DFS (n=32) | |
| --- | --- | --- | --- | --- | --- |
| Age | ≥ 70 vs. < 70 | 0.81 [0.21; 3.1] | 0.76 | 1.82 [0.34; 9.63] | 0.48 |
| Gender | male vs. female | 1.43 [0.25; 8.1] | 0.69 | 0.68 [0.11; 4.27] | 0.68 |
| T stage | T3/4 vs. T2 | 745988151 | 0.9989 | 742903666 |  |
| N stage | N+ vs. N0 | 3 [0.81; 11.19] | 0.1 | 3.31 [0.77; 14.19] | 0.11 |
| OSR2 | median | 4.66 [0.93; 23.35] | 0.06 | 4.3 [0.8; 23.21] | 0.09 |
