# Supplementary material for: A comprehensive molecular characterization of the 8q22.2 region reveals the prognostic relevance of OSR2 mRNA in muscle invasive bladder cancer
Source: PLoS One. 2021 Mar 12;16(3):e0248342. doi: 10.1371/journal.pone.0248342 (PMC7954304; doi:10.1371/journal.pone.0248342)
Supplement: Attachment — Submitted filename: Response to Reviewers .docx [file pone.0248342.s025.docx]

**Response to Reviewers**

*4. Is the manuscript presented in an intelligible fashion and written in standard English?*

The manuscript has been sent to a professional editing service to check and correct any typographical and grammatical errors.

Reviewer #1:

*Nonetheless a possible concern comes from the fact that only TCGA data are used in the analyses. The clinicla annotation of this dataset can be controversial, therefore a possible validation including other cohorts is strictly recommended.*

*In particular the possible validation of the identified genes by RTqPCR in other cohorts is highly relevant.*

We agree with Reviewer #1 that validation in another dataset would increase the validity of our findings. An additional validation using *RT-qPCR* in an institutional cohort could validate, that OSR2 mRNA overexpression (≥ median) significantly predicted decreased OS rates (HR [CI] 6.25 [1.37; 28.38]; p=0.0177) and showed a trend towards decreased DFS rates (HR [CI] 4.54 [0.93; 22.16]; p=0.06). COX6C overexpression (≥ median) was associated with worse OS (HR [CI] 2.6 [0.92; 7.29]; p=0.07) and DFS (HR [CI] 3.05 [0.81; 11.52]; p=0.099). Contrary to our findings in the in silico analysis, OSR2 overexpression (≥ median) was not an independent prognostic factor for DFS (HR [CI] 4.3 [0.8; 23.21]; p=0.09) in the multivariable analysis.

*Another aspect of potential controversial is the association with specific molecular subtypes. According the manuscript the authors observed a possible association with luminal tumors. However in TCGA there are many other subtypes besides luminal and basal BC. Therefore, it is difficult to ascertain how the aithors have performed this analysis. A more detailed study including specific markers is recommended.*

As suggested by Reviewer#1 we extended our analysis of mRNA expression based molecular subtypes. Unfortunately, an annotation of the consensus classification, as described by Kamoun et al., was not available for the TCGA cohort^1^.

As a narrowing we performed a more detailed analysis of five mRNA expression based molecular subtypes, as described by Robertson et al. ^2^

The data shows significantly higher percentages of luminal subtype among AMP patients, and basal squamous subtype in NONAMP patients (S4 Table). Patients with mRNA overexpression in the core amplicon genes RNF19A and SPAG1 show a higher rate of luminal papillary subtype expression.

S4 Table. Subgroup analysis of molecular subtypes in the TCGA cohort.

| molecular subtype | **DNA_Amplicon_Core**  **(% of Amplified and Nonamplified patients with respective subtype)** | | P-value | **RNA_Amplicon_ Core**  **(% of Amplified and Nonamplified patients with respective subtype)** | | P-value |
| --- | --- | --- | --- | --- | --- | --- |
|  | AMP | NONAMP |  | AMP | NONAMP |  |
| luminal | 71 | 57 | **0.04** | 83 | 57 | **0.004** |
| basal squamous | 24 | 39 | **0.03** | 17 | 39 | **0.02** |
| Luminal papillary | 26 | 34 | 0.24 | 50 | 31 | **0.03** |
| Luminal infiltrated | 21 | 19 | 0.77 | 10 | 20 | 0.18 |
| Neuronal | 5 | 4 | 0.69 | 0 | 5 | 0.23 |

In our initial analysis we chose to investigate only the luminal-like and basal-like subtype, as these represent the top of the subtype hierarchy. This is supported by research by Sjödahl et al., who advocate for the initial use of major subtype classes in a proposed algorithm for immunohistochemical (IHC) subtyping of muscle‐invasive bladder cancers^3^.

*The use of a z-score normalization value ≥2 is appropriate but a supplementary table providing values using more or less restrictive z-scores would help to realize the relevance of the amplicon*

To account for a more or less restrictive z-score normalization, z-scores of 1 and 3 were chosen. Based on these z-scores a univariable analysis of the core amplicon for OS and DFS was performed (Table S2). OS was significant (p=0.046) for a z-score of 1. However, this significance could not be maintained in a multivariable analysis (Table S2).

S1 Table. Univariable analysis of mRNA amplicon definitions with different z-score cut-offs.

| z-score | Overall survival | | Disease-free survival | |
| --- | --- | --- | --- | --- |
|  | HR | P-value | HR | P-value |
| **1** | **0.66 [0.42; 1.01]** | **0.046** | 0.93 [0.6; 1.43] | 0.73 |
| 2 | 0.6 [0.32; 1.14] | 0.12 | 1.04 [0.59; 1.82] | 0.88 |
| 3 | 0.72 [0.35; 1.46] | 0.36 | 1.1 [0.58; 2.1] | 0.77 |

Table S2. Multivariable analysis of Overall survival of mRNA amplicon with z-score cut-off z=1.

| **Clinicopathologic variables** |  | Overall survival | |
| --- | --- | --- | --- |
|  |  | HR | P-value |
|  |  |  |  |
| Age | ≥70 vs <70 | 1.76 [1.24; 2.49] | 0.0015 |
| T stage | T3/4 vs T≤2 | 1.5 [0.98; 2.32] | 0.065 |
| Lymph node status | N+ vs N0 | 2.05 [1.44; 2.91] | <0.001 |
| Molecular subtype | (basal vs. luminal) | 1.2 [0.84; 1.7] | 0.32 |
| RNA_Amplicon_Core_z2 | AMP vs NONAMP | 0.65 [0.28; 1.52] | 0.32 |
| RNA_Amplicon_Core_z1 | AMP vs NONAMP | 0.98 [0.54; 1.77] | 0.94 |
| RNA_AMP_COX6C | HIGH vs LOW | 1.31 [0.8; 2.14] | 0.29 |
| RNA_AMP_OSR2 | HIGH vs LOW | 0.71 [0.43; 1.16] | 0.17 |

Reviewer #2:

*1. Introduction: The authors should decide if this is a paper describing novel biological/clinical features associated the 8q22.2 region in bladder cancer – or if this is a more technical paper, describing the use of the methods used. This could be rewritten to focus on one or the other. It is not clear when reading the introduction what the main scope is. The final part of the introduction (line 99) should be rewritten.*

The discovery of new prognostic markers in the 8q22.2 region in bladder cancer resulted from using mRNA expression as a surrogate marker for CN Amplification. Thus, we feel that the main scope of the paper is the use of this method. The final part of the introduction has been modified accordingly.

Text passage:

i) “The primary aim of this study is to provide a method for a comprehensive molecular characterization of the 8q22.2 region in bladder cancer using in silico CNA and mRNA gene expression data.
ii) A second aim of this study is to compare the CNA and mRNA amplicon structure of 8q22.2, investigate the prognostic value of 8q22.2 amplification on survival, and identify predictive genes. Prognostically significant genes are validated using RT-qPCR in an independent cohort of MIBC tumor samples.
iii) Finally, this study is also designed to explore whether the number of chromosomal alterations is associated with a distinct group of patients with clinically or histologically defined parameters.”

*2. The main focus of the paper- chromosomal region 8q22.2 – is mentioned in the introduction as amplified in the [cbioportal.org](http://cbioportal.org) website. However, as little is known about this in BC I suggest to move this to the result section. Overall, 8q22.2 may be interesting, but it is not clear why the authors choose to investigate this. This should be clearly written and expanded upon in the manuscript.*

We do agree with Reviewer #2 that the fact that more than ten percent of MIBC patients showing alterations in 8q22.2 is already a result in and of itself and could thus also be placed in the results section.

However, we chose to report this information in the introduction, because the discrepancy of a high number of patients with MIBC (>10%) showing amplifications in the 8q22.2 region and the largely unexplored role of 8q22.2 in BC, was the primary reason for our investigation of 8q22.2 in MIBC. This was mentioned in the manuscript at page 11.

Text passage:

“More than ten percent of patients in the Cancer Genome Atlas (TCGA) cohort show CNA amplifications within the 8q22.2 region (https://www.cbioportal.org).”

*3. Methods: it is not clear if CNV data is derived from SNP arrays or from exome sequencing. Please specify. Also, as CNV is one of the main points in the manuscript, the authors should describe the different types og gains (high vs low gain, and imbalanced vs balanced). Also – how does the level of amplification affect the level of expression?*

Reviewer #2 is absolutely correct in requesting clarification about the origin of the CNV data. Robertson et al. described the use of Affymetrix SNP6.0 arrays to assess somatic copy number alterations (SCNAs) ^2^.

Text passage:

“CNA data were generated using Affymetrix SNP6.0 arrays [7].”

The level of amplification affects the level of expression in a classical (amplification leading to mRNA overexpression) as well as a paradoxical or contrasting pattern (amplification leading to lower mRNA expression levels compared to non-amplification).

Text passages:

“A classical amplicon definition would suggest an increase in transcript levels in DNA amplified samples. As anticipated, *RNF19A* and *SPAG1* showed a higher median gene expression in the AMP group. A marked difference between both groups could also be observed for *POLR2K*, *COX6C*, and *RIDA*. Interestingly, *RGS22*, *KCNS2*, and *NIPAL2* showed negative gene expression in both groups. Several genes also showed a switch from negative gene expression in the NONAMP group to positive gene expression in the AMP group (Fig 2, S1 Table) ^4^.”

“**Genes of the 8q22.2 region exhibit typical and paradoxical patterns of copy number alterations and RNA expression levels**

While data from breast cancer and other cancers suggest that amplified CNA leads to subsequent mRNA overexpression, our results show that genes of the 8q22.2 region express a heterogenous concordance pattern between CNA and mRNA gene expression levels [29],[31]–[33]. While the classical pattern of CNA amplification leading to mRNA overexpression seems to be conserved in the core region of RNF19A and SPAG1, genes such as KCNS2 and NIPAL2 demonstrate a contrasting pattern, with a higher median gene expression in non-amplified patients. This paradoxical pattern of opposing CNA and mRNA expression levels has been described in lung adenocarcinoma by Tokar et al., who proposed that epigenetic regulatory mechanisms were responsible for these changes [34].”

*4. Are the gains at 8q22.2 associated with other genomic events? This may indicate that this event is an artefact, especially if gains are balanced.*

Initially, we did not assess, whether 8q22.2 gains were associated with any other genomic events except for the 18 mutations analyzed in this study. This analysis was extended to include deletions and amplifications of chromosomal regions (Table S7).

Text passage:

“We further assessed, whether amplification at the 8q22.2 locus was associated with genomic events such as mutations and chromosomal aberrations, including amplifications and deletions.”

*5. Methods: patients receiving NAC are excluded. The reason for this is not clear – the full cohort receive various treatments, and delayed chemotherapy upon metastatic disease, and adjuvant chemotherapy. Please comment on this.*

We thank Reviewer #2 for pointing out the need for clarification for our decision to exclude patients treated with NAC. While the full cohort does indeed receive various treatments, we specifically chose to exclude NAC, because the sampling occurred after radical cystectomy. Thus, NAC could have changed the tumor biology and possibly induced CNA and mRNA changes within the samples. However, postoperative treatment does not change the specimen obtained at RC and does not confound the results obtained from this sample. We addressed the impact of different treatments and prognostic associations in the discussion.

Text passage:

„Associations of chemotherapy resistance and 8q22 amplification have been shown by other groups. Clearly additional experiments are needed to address the potential involvement of 8q22.2 in treatment resistance [48],[49].“

*6. Page 6 – there seem to be redundancy in the text regarding the patient population exclusion criteria.*

Reviewer #2 correctly observes, that the twofold mentioning of the exclusion criteria on page 6 may seem like a redundancy. However, this information appears twice, because the second mention belongs to the legend of figure 1., which has been inserted on this page due to journal specifications.

Text passage:

“Fig 1. REMARK diagram of the study cohort based on the TCGA cell 2017 MIBC cohort

Receipt of neoadjuvant chemotherapy and unknown tumor stage (Tx) or T<2 were defined as exclusion criteria. After exclusion, 357 patients remained for final analysis.”

*7. Methods: how was the z-score of >=2 chosen for mRNA overexpression ? Clarify the definition of “RNA_HIGH_GeneX” and “RNA_LOW_GeneX”.*

The z-score of ≥2 for mRNA overexpression was chosen because it is the default setting on cBioPortal and thus used by many authors when downloading mRNA data from this website. ^5^

We thank Reviewer #2 for pointing out the need for clarifying the definition of “RNA_HIGH_GeneX” and “RNA_LOW_GeneX”. *“*HIGH” and “LOW” refer to the fact that mRNA of a single GeneX is either overexpressed, i.e. z-score of ≥2 or not overexpressed, i.e. z-score of <2.

Gao et al.: “For mRNA or miRNA data and protein and phosphoprotein data, z scores are precomputed from the expression values, and users can specify the threshold or use the default setting (2 SDs from the mean)” ^5^

*8. Methods + Table 2: Why include variables with a p-value < 0.2 in the multivariable analysis?*

We thank reviewer #2 for highlighting the need for an explanation for the use of a p-value < 0.2 in the multivariable analysis. A more liberal cut-off value of 0.2 was chosen to account for relevant parameters on multivariable analysis that would have been missed with a stricter p-value such as <0.05.

Text passage:

“Multivariable analyses were performed using Cox-proportional hazard regression models. For results from the univariable analysis a p value cut-off of <0.2 was chosen to include relevant clinical or pathologic parameters that would have been missed with a more restrictive p value of <0.05 [30]. Variables for the multivariable analysis included significant (p<0.2) clinicopathological characteristics on univariable analysis (pT-Stage, pN-Stage, age, gender,) and genes (RNA_AMP_COX6C, RNA_AMP_OSR2), or amplicon definitions (DNA_Amplicon_Core, RNA_Amplicon_Core).”

*9. Line 184: The authors call a p-value < 0.2 significant. However, they also state in line 192 that p-values < 0.05 were considered significant. Please clarify.*

Reviewer #2 is absolutely correcting in pointing out this discrepancy in our manuscript and the need for clarification. While results were considered significant if a significance level of <0.05 was reached, this level was increased to <0.2 for variables in the univariable analysis. This was done to account for parameters from univariable analysis that might not reach a significance level of <0.05, but might still hold relevance for results obtained on multivariable analysis.

Text passage:

“Results were considered to be significant if significance levels lower than 0.05 were obtained (the exception being significance levels lower than 0.2 on univariable analysis that were included in the multivariable analysis). “

*10. Line 138: the reason for splitting the 8q22.2 into a “core “ and “extended” region is not clear. An explanation is provided in line 146-147, but does this represent a continuous part of DNA at 8q22.2, or simply representation of genes where no correlation between DNA and RNA is observed throughout the region? Furthermore, the results related to this is presented in the method section. (Table 1).*

The reason for splitting the amplicon into a „core” and “extended” region was based purely on the percentage of co-amplification of genes. This approach was applied to heatmaps (oncoprints) of the 8q22.2 region from cBioPortal (Fig S1). This is in line with findings from Kauraniemi et al. and Sahlberg et al. who used a similar approach to define a smallest common region of amplification on the 17q12 amplicon in breast cancer. ^6,7^

*11. Table 2: Luminal vs basal subtype is a strong dichotomization of the data, and it is not strange that this becomes significant. But it would be more interesting to see how the regions actually correlated to the consensus classification for MIBC.*

As suggested by Reviewer#1 we extended our analysis of mRNA expression based molecular subtypes. Unfortunately, an annotation of the consensus classification, as described by Kamoun et al., was not available for the TCGA cohort^1^.

As a narrowing we performed a more detailed analysis of five mRNA expression based molecular subtypes, as described by Robertson et al. ^2^

The data shows significantly higher percentages of luminal subtype among AMP patients, and basal squamous subtype in NONAMP patients (S4 Table). Patients with mRNA overexpression in the core amplicon genes RNF19A and SPAG1 show a higher rate of luminal papillary subtype expression.

S4 Table. Subgroup analysis of molecular subtypes in the TCGA cohort.

| molecular subtype | **DNA_Amplicon_Core**  **(% of Amplified and Nonamplified patients with respective subtype)** | | P-value | **RNA_Amplicon_ Core**  **(% of Amplified and Nonamplified patients with respective subtype)** | | P-value |
| --- | --- | --- | --- | --- | --- | --- |
|  | AMP | NONAMP |  | AMP | NONAMP |  |
| luminal | 71 | 57 | **0.04** | 83 | 57 | **0.004** |
| basal squamous | 24 | 39 | **0.03** | 17 | 39 | **0.02** |
| Luminal papillary | 26 | 34 | 0.24 | 50 | 31 | **0.03** |
| Luminal infiltrated | 21 | 19 | 0.77 | 10 | 20 | 0.18 |
| Neuronal | 5 | 4 | 0.69 | 0 | 5 | 0.23 |

In our initial analysis we chose to investigate only the luminal-like and basal-like subtype, as these represent the top of the subtype hierarchy. This is supported by research by Sjödahl et al., who advocate for the initial use of major subtype classes in a proposed algorithm for immunohistochemical (IHC) subtyping of muscle‐invasive bladder cancers^3^.

*12. Table 2: According to the supplementary excel file, no tumors below T2 are included. However, tumors are divided into T-stage ≤2 and T stage 3/4 in Table 2 and in line 153,201,229. The ≤2 T stage indicates that non-muscle invasive tumors are included and is therefore misleading. Rephrase this to T2 and not ≤T2.*

Thank you for the important comment. Because the TCGA Cell 2017 cohort also contains T1 tumors, our initial analysis also included T1 stage tumors. To make the data more consistent ≤T2 has been rephrased to T2 and the results have been recalculated, as suggested.

*13. N-stage is only significant for RNA and not for DNA, which indicated that the expression is not driven by amplification. In my opinion, it would be stronger to make a joint analysis of genes in the region – affected by amplification, and simultaneously overexpressed. As I read the paper this is not the case, and this is investigated separately. The authors touch upon this in the section starting line 278, but it is not clear why this is not performed in general through the manuscript – if the purpose is to identify genes of interest affected by gene amplification and with elevated expression.*

We thank Reviewer #2 for suggesting a joint analysis of simultaneously amplified and overexpressed genes in the region. We performed an exemplary analysis for the N-stage for the core genes, RNF19A and SPAG1, as well as OSR2 and COX6C to account for different combinations of amplification and overexpression. (Fig S5)

*14. Correlation to mutations and gene functions: the authors correlate to 18 gene mutations. Was correction for multiple testing performed here ? The significance reported is not high, so it would be important to correct for this.*

We thank Reviewer#2 for pointing out the need for correction for multiple testing. While we initially did not correct for multiple testing, this was readjusted using the Bonferroni correction (Fig S6). The manuscript has been changed accordingly.

Text passage:

“Association with mutations and genomic events was investigated using Chi-Square tests, and the Bonferroni correction was used to correct for multiple testing genomic events.”

*15. Finally, the authors should investigate other public datasets, in order to validate the findings – e.g. only RNA data to validate specific genes.*

We agree with Reviewer #2 that our initial findings required further validation. We performed a RT-qPCR analysis of the prognostic genes OSR2 and COX6C in a cohort of 46 radical cystectomy MIBC tumor samples from our institution in order to validate genes using mRNA data only.

References

1.	Kamoun A, de Reyniès A, Allory Y, Sjödahl G, Robertson AG, Seiler R, et al. A Consensus Molecular Classification of Muscle-invasive Bladder Cancer[Formula presented]. Eur Urol [Internet]. 2020 Apr 1 [cited 2020 Sep 17];77(4):420–33. Available from: https://pubmed.ncbi.nlm.nih.gov/31563503/

2.	Robertson AG, Kim J, Al-Ahmadie H, Bellmunt J, Guo G, Cherniack AD, et al. Comprehensive Molecular Characterization of Muscle-Invasive Bladder Cancer. Cell [Internet]. 2018 [cited 2019 Sep 10];174(4):1033. Available from: http://www.ncbi.nlm.nih.gov/pubmed/30096301

3.	Sjödahl G, Jackson CL, Bartlett JM, Siemens DR, Berman DM. Molecular profiling in muscle‐invasive bladder cancer: more than the sum of its parts. J Pathol [Internet]. 2019 Apr 20 [cited 2020 Sep 23];247(5):563–73. Available from: https://onlinelibrary.wiley.com/doi/abs/10.1002/path.5230

4.	Hyman E, Kauraniemi P, Hautaniemi S, Wolf M, Mousses S, Rozenblum E, et al. Impact of DNA Amplification on Gene Expression Patterns in Breast Cancer. Cancer Res. 2002;62(21).

5.	Gao J, Aksoy BA, Dogrusoz U, Dresdner G, Gross B, Sumer SO, et al. Integrative analysis of complex cancer genomics and clinical profiles using the cBioPortal. Sci Signal. 2013 Apr 2;6(269).

6.	Kauraniemi P, Kallioniemi A. Activation of multiple cancer-associated genes at the ERBB2 amplicon in breast cancer. Endocr Relat Cancer [Internet]. 2006 Mar [cited 2020 Mar 19];13(1):39–49. Available from: http://www.ncbi.nlm.nih.gov/pubmed/16601278

7.	Sahlberg KK, Hongisto V, Edgren H, Mäkelä R, Hellström K, Due EU, et al. The HER2 amplicon includes several genes required for the growth and survival of HER2 positive breast cancer cells. Mol Oncol [Internet]. 2013 Jun [cited 2019 Sep 10];7(3):392–401. Available from: http://www.ncbi.nlm.nih.gov/pubmed/23253899
