# Supplementary material for: A comprehensive molecular characterization of the 8q22.2 region reveals the prognostic relevance of OSR2 mRNA in muscle invasive bladder cancer
Source: PLoS One. 2021 Mar 12;16(3):e0248342. doi: 10.1371/journal.pone.0248342 (PMC7954304; doi:10.1371/journal.pone.0248342)
Supplement: Attachment — Submitted filename: Response to Reviewers.docx [file pone.0248342.s026.docx]

**Response to Reviewers**

*1*. *line 126: I think the use of "in silico" i headline and in line 127 is a bit strange. I would rewrite and just indicate that data has been produced earlier*

We thank Reviewer #2 for his suggestion. The headline and line 127 have been modified accordingly.

Text passage:

**“TCGA cohort**

Data for the first muscle invasive bladder cancer (MIBC) cohort for this study was derived from The Cancer Genome Atlas and has been produced in earlier analyses [7].”

*2. line 152: rewrite to just mention a validation cohort - it is not important to highlight the origin in the headline*

The headline has been modified.

Text passage:

“Validation cohort”

*3. line 284: remove the "cell 2017" from the headline - and "the" before TCGA as it is already included in the acronym..*

The headline has been modified according to the suggestions or Reviewer #2.

Text passage:

“8q22.2 is a highly amplified region in TCGA cohort and can be divided into a core and extended region “

*4. line 325: remove "in silico" again & 5.  line 382: remove "in silico"*

We thank Reviewer #2 for his suggestions. “In silico” has been omitted in the text passages.

Text passage:

“Univariable and multivariable survival analysis (DFS and OS) of the 8q22.2 region and genes in the TCGA cohort”

“Validation of the biomarkers COX6C and OSR2”
